# Mechanotransduction in talin through the interaction of the R8 domain with DLC1

**DOI:** 10.1371/journal.pbio.2005599

**Published:** 2018-07-20

**Authors:** Alexander William M. Haining, Rolle Rahikainen, Ernesto Cortes, Dariusz Lachowski, Alistair Rice, Magdalena von Essen, Vesa P. Hytönen, Armando del Río Hernández

**Affiliations:** 1 Cellular and Molecular Biomechanics Laboratory, Department of Bioengineering, Imperial College London, London, United Kingdom; 2 Faculty of Medicine and Life Sciences and BioMediTech, University of Tampere, Finland and Fimlab Laboratories, Tampere, Finland; University of Münster, Germany

## Abstract

The mechanical unfolding of proteins is a cellular mechanism for force transduction with potentially broad implications in cell fate. Despite this, the mechanism by which protein unfolding elicits differential downstream signalling pathways remains poorly understood. Here, we used protein engineering, atomic force microscopy, and biophysical tools to delineate how protein unfolding controls cell mechanics. Deleted in liver cancer 1 (DLC1) is a negative regulator of Ras homolog family member A (RhoA) and cell contractility that regulates cell behaviour when localised to focal adhesions bound to folded talin. Using a talin mutant resistant to force-induced unfolding of R8 domain, we show that talin unfolding determines DLC1 downstream signalling and, consequently, cell mechanics. We propose that this new mechanism of mechanotransduction may have implications for a wide variety of associated cellular processes.

## Author summary

Mechano-induced conformational changes and the unfolding of protein domains are cornerstones of mechanotransduction and regulate the interaction of proteins with other molecules. Talin is a prominent molecule in focal adhesions and one of the few proteins that simultaneously connects integrin receptors in the cell membrane with the actin cytoskeleton. This bridging position, owing to the cytoskeleton’s contractile nature, exposes talin to forces along its length. In this work, we studied the implications of the R8 domain unfolding in the downstream activity of deleted in liver cancer 1 (DLC1), which binds the talin R8 domain and negatively regulates Ras homolog family member A (RhoA). We created a talin mutant with the R8 domain resistant to mechanical unfolding and observed that cells expressing these talin mutants have altered patterns of focal adhesion dynamics and lower levels of actomyosin contraction. This leads to decreased traction forces and diminished cell migration. We propose a novel force-controlled molecular switch that refines the mechanism of talin-mediated focal adhesion activation, providing negative feedback during focal adhesion maturation. The broader effects of this talin-mediated mechanism need to be elucidated, as it might regulate multiple cellular events.

Mechanical force is increasingly being accepted as a key modulator of cellular behaviour. These forces can be applied externally from the extracellular matrix (ECM) or internally generated from the active actin cytoskeleton pulling against a cell’s attachment to the ECM. Unlocking the mechanisms by which cells sense and translate these forces (mechanotransduction) is an active area of research, given its wide-ranging implications. The mechanically induced unfolding of mechanosensitive proteins has provided a new mechanism for force transduction. The disruption of the tertiary structure of these proteins can lead to the exposure of cryptic sites for ligand binding, molecule activation, or cleavage of domains by proteases [1–3]. However, there is still a gap in our understanding of how this mechanically induced protein unfolding triggers distinctive, or alternative, large-scale downstream signalling in cells to differentially control their behaviour. Here, we used single-molecule techniques, combined with protein engineering and cellular biophysical approaches, to characterise how talin mechanotransduction through the interaction of the R8 domain with deleted in liver cancer 1 (DLC1) can regulate cell mechanics.

**Fig 1:**
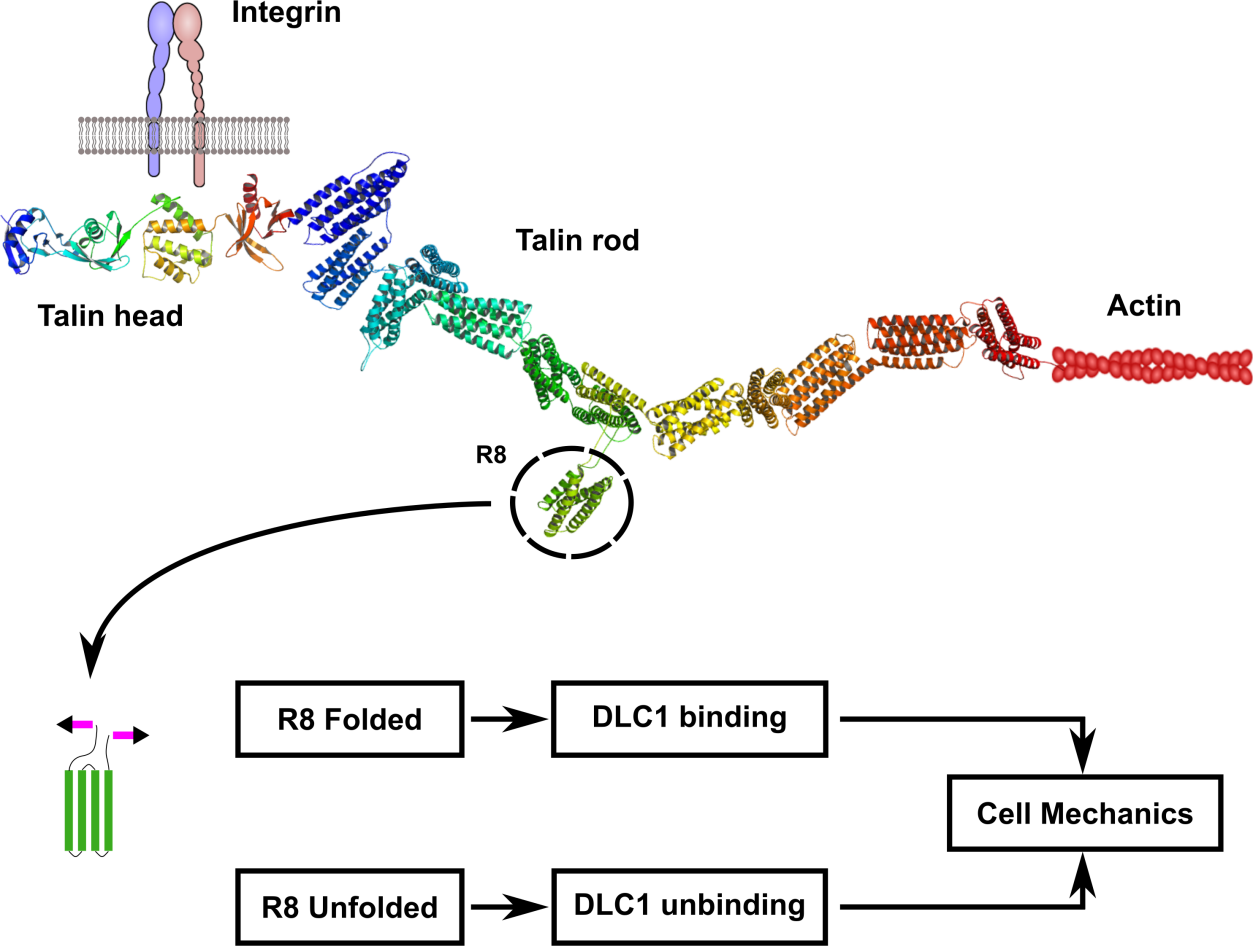
The unfolding/folding state of the R8 region of the talin rod modulates cell mechanics through DLC1 recruitment to focal adhesions. Talin simultaneously links integrins with the actin cytoskeleton. The R8 region protrudes out of the talin molecule structure, and a disulphide bridge was added to the linker area of R7-R8 to prevent R8 unfolding under mechanical load. The folded R8 region promotes DLC1 binding to talin and localisation to focal adhesions. This activates the negative regulation of the RhoA pathway that decreases myosin activation, actomyosin contractility, force generation, and migration. On the other hand, force application on the native talin molecule would unfold the R8 region producing the unbinding and deactivation of DLC1, which does not localise to the focal adhesion and loses its capacity to regulate the RhoA pathway. DLC1, deleted in liver cancer 1; RhoA, Ras homolog family member A.

Talin, one of the most studied mechanosensitive molecules, is a key protein in focal adhesions, where it simultaneously connects integrins in the cell membrane through its N-terminal head domain and the actin cytoskeleton using the C-terminal rod (Fig 1). This bridging position, owing to the cytoskeleton’s contractile nature, exposes talin to forces along its length. This tension can subsequently unfold talin domains, changing its affinity for binding partners. Most of the work on talin’s mechanosensitivity has focussed on the regions immediately after the head domain—namely, regions R1 to R3—and on its interaction with vinculin, another important structural and signalling focal adhesion protein [1, 4, 5]. Cellular studies utilising Förster resonance energy transfer (FRET) sensors have measured the tension that a single talin linkage experiences [6, 7]. Alongside this, our recent work has demonstrated that this level of tension is sufficient to unfold significant portions of the alpha-helical bundled structure of the talin molecule, in addition to R1-R3, that are between its integrin- and actin-binding sites[8].

These findings open a plethora of possibilities to explore how talin interacts with other molecules to control cell function, given the wide range of proteins that interact with the talin rod. DLC1—a Ras homolog GTPase-activating protein (RhoGAP) that negatively regulates the activity of RhoA-C and is a potent tumour suppressor whose loss has been implicated with numerous cancers [9, 10]—is a notably interesting talin-binding partner. In order for its regulatory role on the Rho family of GTPases to have an impact on cellular function, DLC1 requires to be localised in focal adhesions, and this localisation is mediated by its interaction with talin [11]. DLC1 binds to the R8 helical bundle of the talin rod. This bundle is unique in terms of molecular connections—it does not lie in the chain between two other bundles in the talin rod but exists rather as a protrusion of the R7 bundle (Fig 1). The nature of DLC1’s binding to R8 is such that it forms an interaction with two of the helices in R8, effectively creating a 5-helix bundle out of R8’s 4 helices and one of DLC1’s [12]. This proposed mechanism of interaction suggests that if the R8 bundle structure is disrupted, it would abrogate the binding to DLC1.

Structural in vitro and cellular studies have indicated that such disruption is likely to occur as part of the cell’s cycle of adhesion formation and maturation. Previous studies have measured both the length of the talin protein in cells and the internal tension of the rod [6, 13, 14]. The extension of the molecule varies between 50–350 nm, which would necessitate the unfolding of multiple talin rod subdomains. These extension data were also combined in a previous study with single-molecule data to provide a model of talin subdomain unfolding in cells [15]. This model demonstrated that intramolecular tension, measured in cells at 5–10 pN, is maintained by the dynamic unfolding and refolding of talin rod subdomains. At the higher extensions of the rod, as many as 9 of the 12 α-helix subdomains are unfolded. The R7-R8 bundle is intermediate in stability with respect to the rest of the rod and is likely to unfold over the order of a few seconds at physiological loading rates [8, 15]. Given that myosin-dependent stretching of talin occurs over 6–16 s [14], it is likely that a significant proportion of R7-R8 bundles are disrupted, especially as adhesions mature and develop more stable actin linkages. We hypothesised that unfolding of the R8 region of talin by intracellular tension may modulate DLC1’s recruitment to focal adhesions mediated by talin; and this triggers differential downstream pathways in cells (Fig 1).

**Fig 2:**
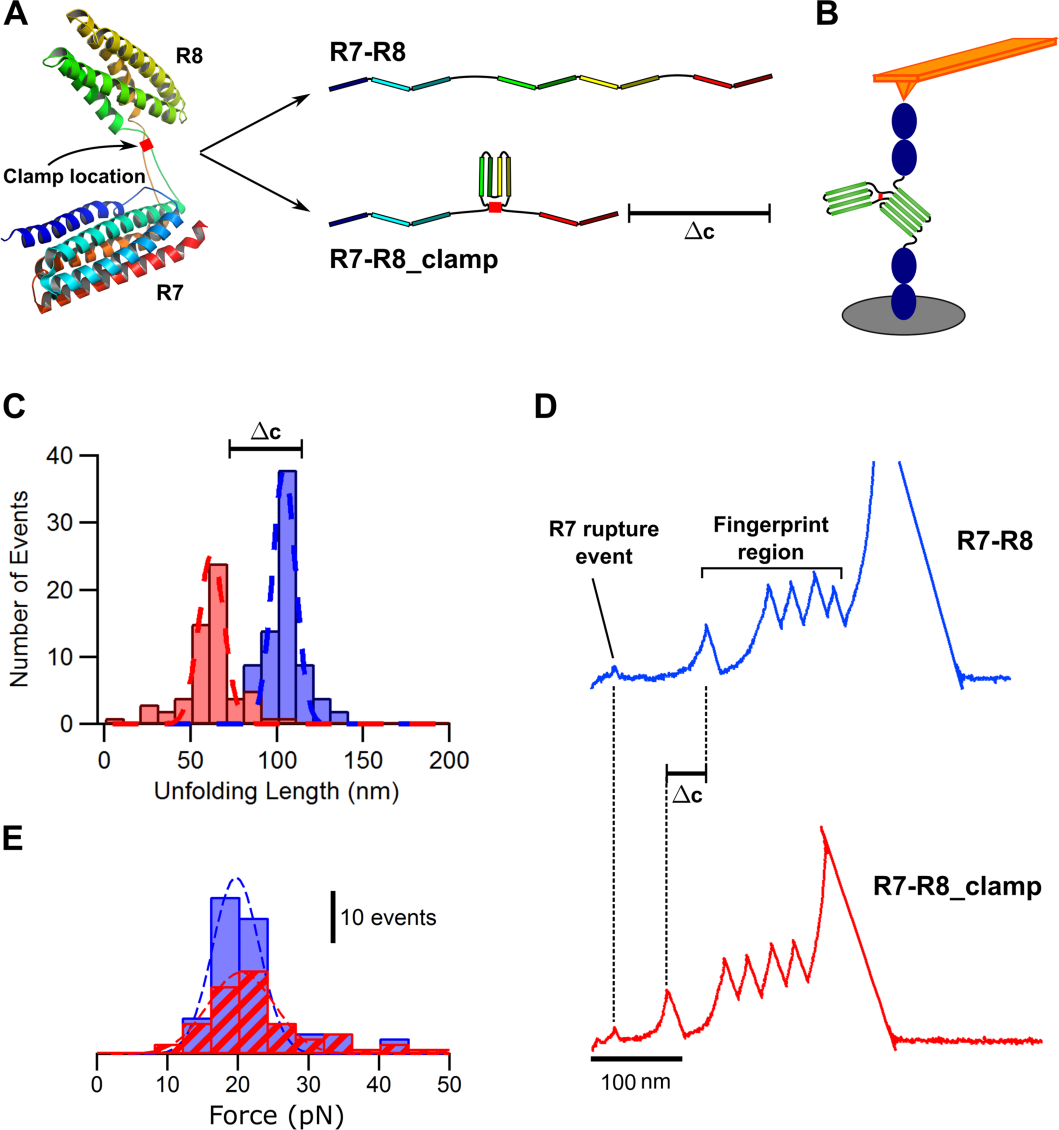
The disulphide clamp inserted in the R8 region of talin prevents R8 subdomain unfolding. (A) Representation of the R7-R8 talin fragment showing the location of the disulphide clamp and how the clamp leads to a difference in unfolding length (ΔC). (B) Schematic of smAFM setup with construct (R7-R8 flanked by fingerprints) extended while trapped between a cantilever and piezo-driven surface. (C) Histogram showing the typical unfolding lengths of the WT R7-R8 (blue) and R7-R8 clamp (red) subdomains. (D) Representative traces highlighting the shortened unfolding pattern of the R7-R8 (blue) and R7-R8_clamp (red) subdomains. (E) Histograms of the unfolding force for R7-R8 (blue) and R7-R8_clamp (red) subdomains. Blue and red dashed lines show gaussian fits applied to the histograms. For panels C and E, *n* = 82 and *n* = 56 traces for R7-R8 and R7-R8_clamp, respectively, taken in more than 5 different experiments. smAFM, single-molecule atomic force microscopy; WT, wild-type.

To experimentally test this hypothesis, we engineered 2 polyproteins amenable to mechanical manipulation using single-molecule atomic force microscopy (smAFM). The first polyprotein (R7-R8) contained the region R7 of the talin molecule and its linked R8 bundle flanked by two units of I27 domains and a HaloTag, as previously reported [8]. Next, we designed a mutant of the R7-R8 subdomain (R7-R8_clamp) to make the R8 bundle resistant to tension-induced unfolding. To do this, we took advantage of the unique structure of the R8 bundle and its attachment to the rest of the talin rod. The long linkers that connect the opposite ends of the R8 bundle’s amino acid chain to the R7 bundle are in close proximity to one another (<5 Å between the backbone carbons) [16]. We hypothesised that the introduction of a cysteine residue into each linker (by single-point mutation to residues Q1459 and S1583) would create a disulphide bridge between the linkers, creating a clamp to hold the R8 bundle together (Fig 2A). The advantage of the use of this R8 mutant is that it will allow the study of the talin unfolding without abrogating binding sites or removing entire subdomains.

We used a force extension protocol, as previously described, to study the unfolding of both polyproteins (Fig 2B) [8]. The wild-type (WT) R7-R8 form unfolds in a single step, with a total unfolding length of 104 ± 7 nm, which represents the unfolding length of both bundles. The clamped R8 mutant, by contrast, shows a reduced length of unfolding of 62 ± 6 nm (Fig 2C and 2D and S1 Data). The difference in unfolding length of around 40 nm matches the expected difference from lack of unfolding of the R8 bundle (124 amino acids’ removal should result in a 38–50 nm reduction in unfolding length). This confirms that the disulphide clamp introduced in the R8 mutant makes this construct resistant to force-induced unfolding. We also observed that the introduction of the clamp did not significantly alter the unfolding forces of the R7-R8 and R7-R8 clamp domains, as both unfold at forces close to 20 pN (Fig 2E and S1 Data).

**Fig 3:**
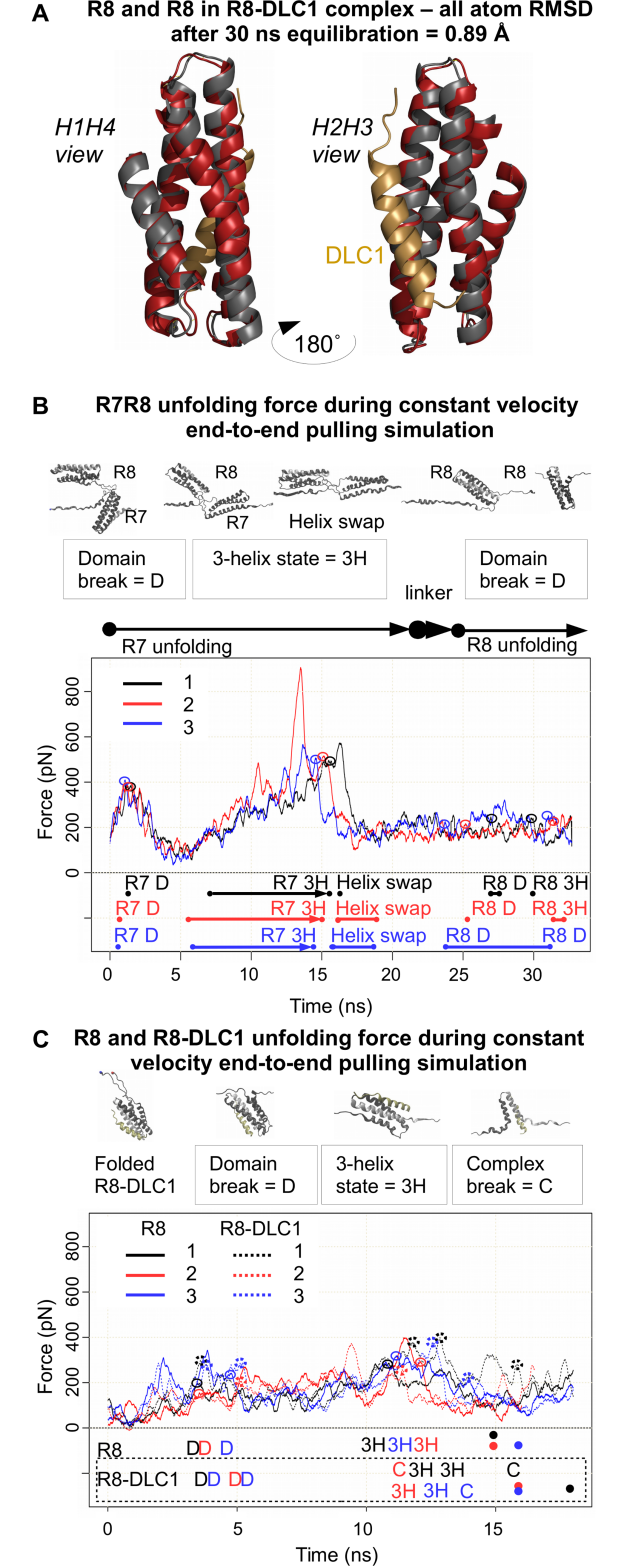
Unfolding of R7-R8, R8 and R8-DLC1 under mechanical load in SMD simulations. (A) Structural alignment of R8 domain (red) and R8 domain in R8-DLC1 complex (grey) (DLC1 –yellow). (B, C) Unfolding force traces in 3 repetitions, examples of structural changes during the domain unfolding and details of the unfolding events over simulation time; B: R7-R8 double domain, C: R8 domain and R8-DLC1 complex. ‘D’ = folded domain, ‘3H’ = 3-helix intermediate, Helix swap state = R8-R7H1 complex, ‘C’ = R8 H2H3-DLC1 complex, ‘dot’ = unfolded domain. DLC1, deleted in liver cancer 1; RMSD, root-mean-square deviation.

We also examined the stability of the R7-R8 domain using steered molecular dynamics (SMD) simulations, as well as investigating the effect of DLC1 binding (Fig 3A) on the stability of the R8 bundle. Previous work has found that 5-helix bundles, such as R7, exhibit higher stabilities than 4-helix bundles, such as R8 [8, 15]. Our SMD analysis with 2 nm/ns constant velocity pulling showed this is the case for the interlinked R7-R8 domain: R7 unfolds first over approximately 15 ns, requiring at least 500 pN of peak force to disrupt the full bundle structure (Fig 3B). After the complete unfolding of the R7 bundle, tension is then transferred to the R8 bundle, which unfolds at peak forces less than 300 pN. This relative instability of the R8 bundle as compared to R7, combined with R7 needing to be unfolded completely before R8 is exposed to tension, leads us to hypothesise that R8’s insertion into R7 regulates its unfolding, and thus binding, characteristics.

Given that the binding of DLC1 to R8 takes the form of a ‘5-helix’ bundle arrangement [12], we examined whether the binding of DLC1 alters the stability of the R8 bundle. SMD analysis of the combined DLC1/R8 and R8 alone showed that this interaction does not significantly alter the unfolding characteristics of the bundle (Fig 3C). This is likely due to the nature of the pulling vectors: despite the contribution of an additional helix from DLC1, R8 is still opening in the ‘zipper’ fashion as opposed to the ‘sliding’ mechanism that has been postulated as the cause of the higher stability of 5-helix bundles (S1 Fig). These SMD results support the smAFM results and the hypothesis of a mechanically weak R8 bundle whose unfolding characteristics are significantly altered through the addition of the disulphide clamp. More detailed analysis of the SMD data can be found in S1 Text.

We then expressed full-length talin constructs, both WT and with the disulphide clamp (R8), inserted within mammalian vectors, in talin-null mouse embryonic fibroblasts (MEFs) [17], to determine the cellular effects of preventing R8 unfolding. The redox potential inside the cell is not homogeneous and may vary depending on the culture conditions, and consequently, we acknowledge the possibility of low levels of cytosolic disulphides [18]. However, a recent study reported a significantly higher oxidative environment inside cells close to the plasma membrane compared to the inner cytoplasmic milieu, which would favour the disulphide clamp stabilisation in the talin molecule [19].

**Fig 4:**
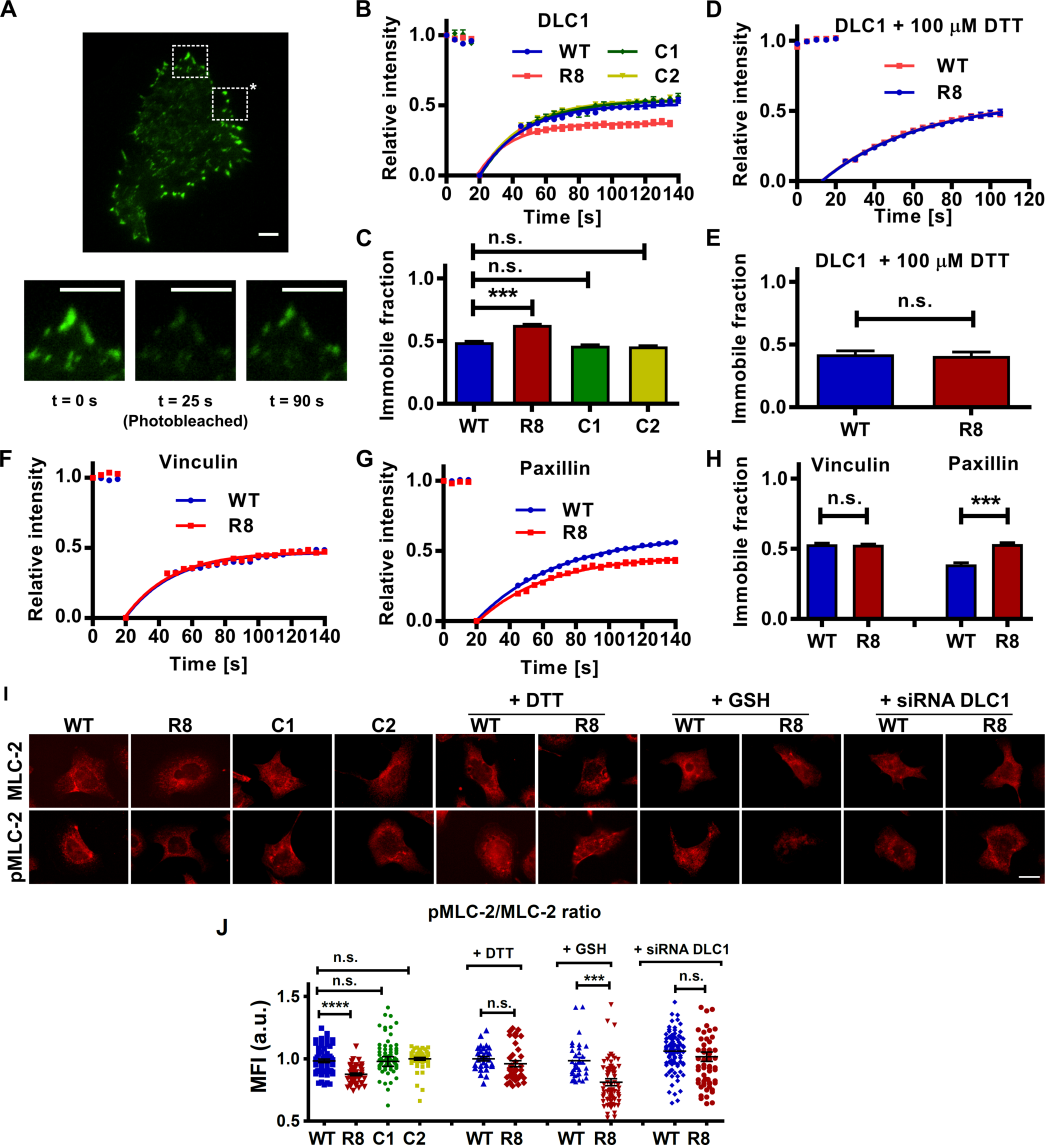
Preventing R8 unfolding increases DLC1 binding and downstream deactivation of MLC-2. (A) Representative images of a cell expressing GFP-DLC1. Insets show the intensity of the focal adhesions at different time points of the FRAP experiments. Region highlighted with asterisk was not exposed to confocal laser (see S2 Fig). Scale bars are 5 μm. (B) FRAP curves for the recovery of GFP-tagged DLC1 for MEFs transfected with either WT talin (‘WT’), the clamped R8 mutant (‘R8’), construct 1 (‘C1’), or construct 2 (‘C2’). (C) Immobile fraction data obtained from fit of FRAP curves (*n* = 25, 32, 26, 28 cells for WT, R8, C1, and C2, respectively). (D) FRAP curves for MEFs treated with DTT to cleave the disulphide clamp showing no difference in recovery. Errors bars represent sem. (E) Immobile fraction data from fit of FRAP curves in D (*n* = 14 cells for both WT and R8). (F-G) FRAP curves for the recovery of GFP vinculin (F) or GFP paxillin (G) in MEFs transfected with either WT talin (WT) or the clamped R8 mutant. (H) Immobile fraction data obtained from fit of FRAP curves in F-G (*n* = 33, 29, 25, 27 cells for WT/vinculin-GFP, R8/vinculin-GFP, WT/paxillin-GFP, R8/paxillin-GFP). (I) Immunofluorescence images of MLC-2 and pMLC-2 staining. Scale bar is 20 μm. (J) Ratio of pMLC-2 staining to total MLC-2 for MEFs transfected with WT talin, the clamped R8 mutant, C1, or C2 constructs. Each dot represents a cell; horizontal lines are mean ± sem. For all panels, histograms represent mean ± sem. All experiments were run in triplicate (*t* test). a.u., arbitrary unit; DLC1, deleted in liver cancer 1; DTT, dithiothreitol; FRAP, fluorescent recovery after photobleaching; GFP, green fluorescent protein; GSH, glutathione; MEF, mouse embryonic fibroblast; MFI, mean fluorescence intensity; MLC-2, myosin light chain 2; n.s., not significant; pMLC-2, phospho-myosin light chain 2; siRNA, small interfering RNA; WT, wild-type.

We first tested the dynamics of focal adhesion–associated DLC1 using fluorescent recovery after photobleaching (FRAP). Along with the talin constructs, the cells were transfected with DLC1 tagged with green fluorescent protein (GFP). A region of the cell containing focal adhesions was then photobleached using a confocal laser. The recovery of the GFP signal into the focal adhesions of the bleached region serves as a measure of the dynamics of the DLC1 in those adhesions (Fig 4A). The recovery curves of the GFP signal show a difference in the immobile fraction between WT and R8 mutant (Fig 4B and S1 Data). Analysis of these curves displays a larger population of immobile DLC1 in the R8 mutant as compared to WT (Fig 4C and S1 Data). This implies a lower turnover of DLC1 in focal adhesions where the R8 bundle is unable to unfold and thus favours DLC1-binding. We created 2 talin constructs, each one harbouring a cysteine residue. These constructs were used as negative controls for our talin R8 clamp, construct C1 with the cysteine residue in position 1459, and construct C2 with the cysteine residue in position 1583. We observed no significant differences in the recovery rates of the GFP signals or in the immobile fraction of DLC1 between cells transfected with either WT, C1, or C2 talin (Fig 4B–4D and S1 Data).

To ensure that this effect was due to the presence of a disulphide bridge in the R8 bundle, we also conducted the FRAP experiment in the presence of dithiothreitol (DTT), a cell-permeable small molecule that reduces intracellular disulphide bonds and thus would disrupt the clamp in subdomain R8. When the FRAP experiments were performed in the presence of DTT, we saw no difference between the recovery curves (Fig 4D and S1 Data), with no significant differences between the immobile fractions as compared to cells expressing WT talin (Fig 4E and S1 Data).

In order to examine if the differences observed in the DLC1 focal adhesion dynamics are present in other focal adhesion proteins, we transfected the MEF cells with either the WT or the R8 talin constructs and GFP vinculin or GFP paxillin. We used the same FRAP protocol and observed no differences in the recovery rates or in the population of the immobile fraction of GFP vinculin between cells expressing WT or R8 (Fig 4F–4H and S1 Data). This is not surprising given that there are 11 vinculin binding sites in the entire talin rod [14], and any potential effect in the binding of vinculin to the R8 domain may be masked or compensated by the entire pool of bound vinculin molecules. In stark contrast, we observed a significant lower recovery rate and significantly larger population of GFP paxillin for MEFs transfected with R8 talin compared to WT cells (Fig 4G and 4H). The recovery curves for paxillin demonstrate a similar difference between WT and R8 as with DLC1. The immobile fraction is greater in R8, again indicating that the disulphide-protected folding of R8 leads to a lower turnover of paxillin (Fig 4H). Paxillin is thought to bind to R8 in an analogous manner to DLC1, with a ΔR8 talin mutant demonstrating reduced levels of paxillin in focal adhesions similar to the reduction in DLC1 [12]. These results indicate that the disruption of the R8 domain abrogates paxillin binding as well as that of DLC1.

Given that the localisation of DLC1 to focal adhesions has been shown to affect downstream cell processes [11], we investigated the effect of the R8 mutant on potentially dependent pathways. Myosin light chain 2 (MLC-2) is downstream of RhoA signalling, and its activation (phosphorylation of serine and threonine residues in the active form—phospho-myosin light chain 2 [pMLC-2]) is proportional to the level of active RhoA [20]. We expected that the greater association of DLC1 with talin in the R8 mutant would down-regulate the activation of RhoA and the downstream effector MLC-2. We used immunofluorescence to quantify the pMLC-2/MLC-2 levels as a surrogate of MLC-2 activation (Fig 4I). We saw a significantly higher pMLC-2/MLC-2 ratio in the WT talin–expressing cells as compared to those with the clamp (R8), indicating greater activation of MLC-2 (Fig 4J, S5 Fig and S1 Data). This difference was abrogated by the addition of DTT but maintained in the presence of glutathione (GSH), a cell membrane–impermeant reducing agent[21]. Furthermore, knocking down DLC1 via small interfering RNA (siRNA) also abolished the differences in the levels of MLC-2 activation between WT and R8 cells. There were no significant differences in the pMLC-2/MLC-2 ratio between WT cells and the cells transfected with either C1 or C2 talin constructs. Taken together, these results show that the presence of the disulphide clamp that makes the R8 region resistant to unfolding and allows DLC1 localisation to focal adhesions was necessary to observe the large-scale downstream effect (Fig 4J).

**Fig 5:**
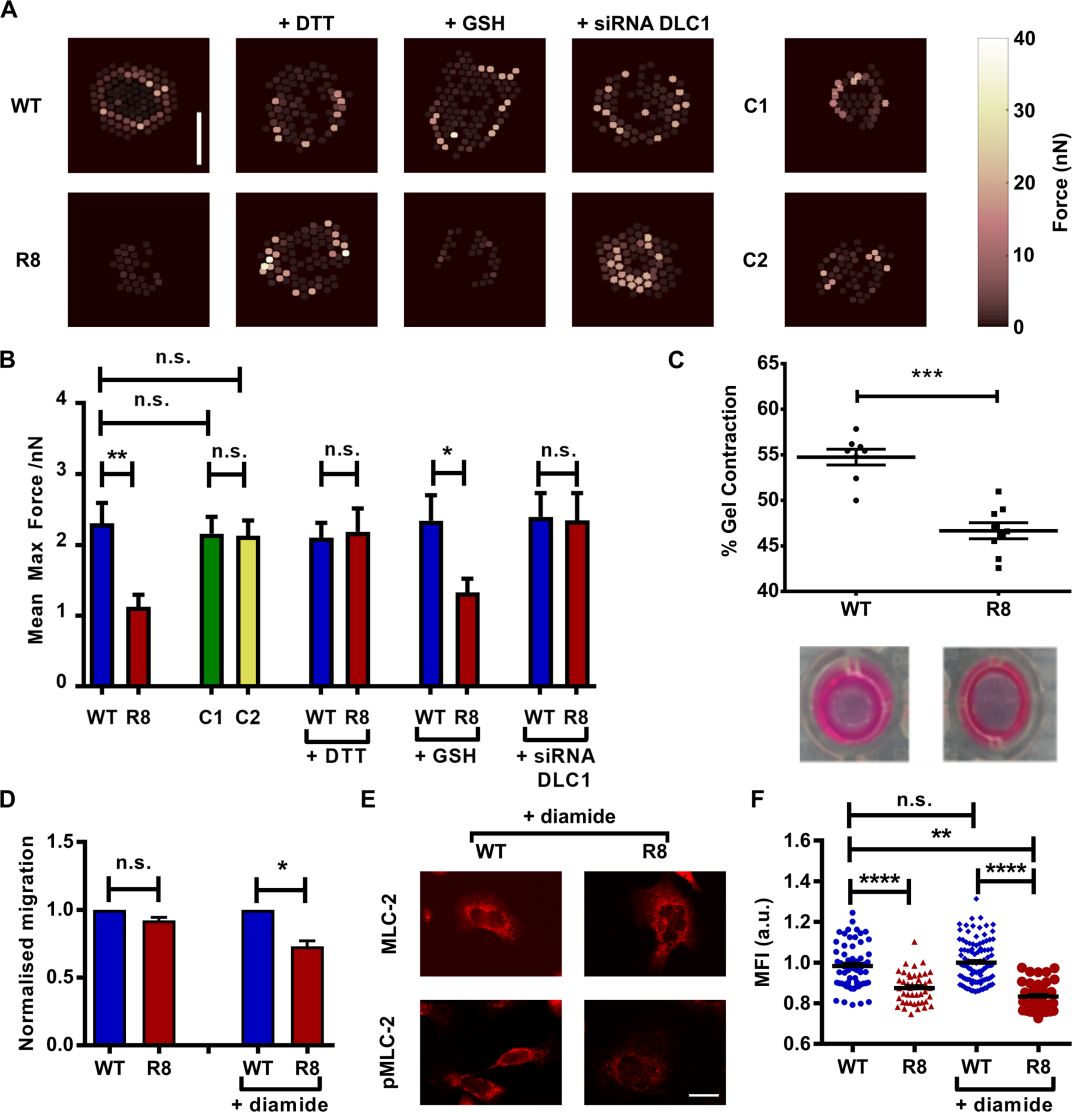
Preventing R8 unfolding reduces force application and cell migration. (A) Heat maps representing forces applied by MEFs on top of the pillars, scale bar = 10 μm. (B) Quantification of average forces applied by MEFs on pillars. (number of analysed cells: 20 WT, 19 R8, 22 C1, 20 C2, 29 WT DTT, 25 R8 DTT, 22 WT GSH, 19 R8 GSH, 27 WT siRNA DLC1, 28 R8 siRNA DLC1). (C) Scatter plot of the percentage change in gel size for collagen Matrigels embedded with transfected MEFs for 48 h (*t* test). (D) Normalised migration rate over 12 h for transfected MEFs, with and without diamide to enhance the disulphide clamp. (Number of cells: 207 WT, 157 R8, 130 WT + diamide, 142 R8 + diamide, *t* test). (E) Immunofluorescence images of MLC-2 and pMLC-2 staining for diamide-treated cells. Scale bar is 20 μm. (F) Ratio of pMLC-2 staining to total MLC-2 for transfected cells, with and without diamide. Each dot represents a cell; horizontal lines are mean ± sem (*t* test). For all panels, histograms represent mean ± sem. All data set in S1 Data. a.u., arbitrary unit; DLC1, deleted in liver cancer 1; DTT, dithiothreitol; GSH, glutathione; MEF, mouse embryonic fibroblast; MFI, mean fluorescence intensity; MLC-2, myosin light chain 2; n.s., not significant; pMLC-2, phospho-myosin light chain 2; siRNA, small interfering RNA; WT, wild-type.

Higher MLC-2 activation is associated with greater cellular traction force generation [22], and so we next sought to examine the effect of the R8 mutant on generating endogenous forces. We seeded cells expressing either WT, R8 mutant, C1, or C2 talin constructs on top of a substrate consisting of an array of flexible elastic polydimethylsiloxane (PDMS) micropillars coated with fibronectin. The deflection of the pillars is optically monitored and is proportional to the forces applied by the cells on the pillars. We observed that cells expressing WT talin applied significantly higher endogenous traction forces in comparison with the group of cells expressing the R8 mutant talin during the 90 minutes of cell spreading (Fig 5A and 5B and S1 Data). There were no significant differences between the forces applied by cells expressing WT, C1, and C2 talin constructs. The observed differences between the forces applied by WT talin and R8 talin cells were maintained in the presence of the cell-impermeant reducing agent GSH but abrogated when the cell-penetrating reducing agent DTT was added or when the cells were previously transfected with siRNA for DLC1. These data corroborate our previous observations on the MLC-2 activation in cells transfected with WT and R8 talin constructs.

The capacity of cells to apply forces on their substrates correlates with their ability to contract collagen gels and to remodel the ECM [23, 24]. To examine the ability of both groups of cells (containing WT and R8 talin) to deform matrices, we embedded the cells within collagen Matrigel gels and observed that after 48 h, the WT cells contracted the gels to 47% of their initial size, whereas the mutant cells only contracted them to 55% of their initial size. This agrees with the previous results that showed that R8 cells displayed significantly lower traction forces (Fig 5C and S1 Data).

The localisation of DLC1 to adhesions has also been shown to negatively affect the ability of the cells to migrate [11]. The migration speeds of the WT and R8 mutant cells were measured over 12 h, revealing a nonsignificant decrease in migration rate between WT and mutant (Fig 5D). We treated the cells with 50 μM diamide to shift the redox balance towards the oxidative range in the cellular cytoplasm so that more of the mutant protein would exhibit the disulphide bond preventing R8 unfolding and DLC1 unbinding. When the migration speed of these cells was tested, we saw a significant decrease in migration speed in the cells containing the R8 mutant in comparison to WT (Fig 5D, S1 Video, and S1 Data). As a further test of the efficacy of diamide in enhancing the effect of the disulphide clamp, we conducted the pMLC-2/MLC-2 staining for cells treated with diamide. In this case again, we saw that the diamide increases the effect, with a greater decrease in pMLC-2/MLC-2 ratio for diamide-treated R8 mutant cells than for those untreated (Fig 5E and 5F, and S1 Data). A 10 μM diamide concentration had clear effect in this assay. We also confirmed the oxidative effect of the diamide for the disulphides by treating WT MEF cells with diamide and then analysing the cell lysate with 2D electrophoresis (S6 Fig), comparing oxidising and reducing conditions as described in a previous study [18].

From these results, we have shown how DLC1 localisation to focal adhesions is modulated by the unfolding of talin’s R8 domain, constituting a novel force-controlled molecular switch. Through stabilising the R8 bundle in the talin molecule with a disulphide bridge, we altered the dynamics of focal adhesion–associated DLC1 (Fig 4B–4E). This led to downstream reductions in MLC-2 activation (Fig 4I and 4J), force generation (Fig 5A and 5B), and cell migration (Fig 5D).

In the widely accepted model of talin-mediated focal adhesion activation, as force is applied to talin in maturing adhesions by the actin contractile machinery, its helical bundles are disrupted. This allows vinculin to bind, which leads to focal adhesion maturation [4, 25]. It also, based on these findings, disrupts the binding of DLC1, reducing its negative effects on RhoA activity, which enhances the generation of forces at growing adhesions. Thus, in early adhesions, this works cooperatively with the increase in vinculin binding to mature the adhesions. However, the weakness of the model as it stood, with the vinculin-talin interaction at its core, is that it failed to account for any form of negative feedback. As the adhesions grew, talin recruited more vinculin, which increased force generation, which led to greater recruitment of talin and vinculin and so on [26].

Our new model provides a further refined mechanism of talin-mediated focal adhesion activation that works synergistically with its binding to vinculin initially but has a more important role to play in mature adhesions. This is where the mechanical switch involving DLC1 does provide a negative feedback and thus control. In the maturing adhesions, it is as before: force on talin unfolds the R8 domain, reducing DLC1 localisation and increasing RhoA activity. However, as the adhesion matures and recruits more proteins to the complex, the force on individual talin molecules is reduced. This is because, although the total force applied to the adhesion is likely increasing, the presence of an increasing number of mechanical linkages, such as talin and vinculin, means that the force on each linkage is reduced, like springs in parallel. The reduction in force on each linkage has been demonstrated in cellular experiments in which the force on linkages has been measured by FRET tension sensors and shown to be lower in more mature, central adhesions than earlier, peripheral ones [7, 27]. This reduction in force on talin means that our R8 switch now reverts to DLC1 binding. This hypothesis is supported by studies showing a greater localisation of DLC1 to mature adhesions [28].

This reduces force generation and controls the maturation process of the adhesions. This is an attractive model not just in light of the results presented but also when considered with respect to the role of DLC1 deletion in cancers. The role of highly activated and contractile cells in the cancer progression has been investigated before, and it is possible that DLC1’s role in cancer (by its absence) is mediated through the subsequent lack of control over focal adhesion activation.

Moreover, when cells are surrounded by stiffer substrates (such as tumours), the talin molecule (anchored to a stiff surface via integrin receptors) would unfold more readily. As can be seen in these results, this will trigger DLC1 deactivation and increased cell contractility, force generation, and migration. Increasing cell contractility in cancer-associated fibroblasts enhances tumour growth via paracrine signalling with the tumour microenvironment [29], and increasing contractility in cancer cells will facilitate the breaching of the basement membrane that surrounds tumours [30]. Furthermore, increasing migration in cancer cells will favour cancer cell invasion. Indeed, the talin molecule has been implicated in diseases that are influenced by mechanical factors, including cancer [31].

The broader implications of the downstream cascades of events resulting from the presented talin mechanotransduction mechanism for the biology of cells need to be established. For instance, this mechanism could affect actomyosin-dependent fibronectin fibrillogenesis, exerting a direct impact on the regulation of connective tissue homeostasis in health (wound healing) and disease. A fibrotic matrix is a major clinical hallmark of several disorders, such as cirrhosis and pancreatitis, often associated with aberrant mechanotransduction, and this mechanism may provide a potential mechano-therapy target.

As well as these results demonstrating the existence of force-mediated binding between talin and DLC1, it also lays out a method for further investigations of talin or other mechanosensitive proteins. Previously, studies of these types of proteins focussed on the removal of subdomains, whereas here we report a methodology to force these mechanical switches into a particular state. Small differences can also be amplified by the addition of reagents that alter the redox state of the cells to favour the fixed state. In this way, we can establish the local effects of these switches along with the downstream phenomena they control. The regulation of signalling pathways by modulating protein structure and ligand binding may be a cellular adaptation cells have developed for rigidity sensing. Further studies utilizing other mechanosensitive proteins would validate this.

## Materials and methods

### Generation of polyprotein constructs and smAFM

The smAFM experiments were carried out according to protocols described previously [8]. The talin fragment polyprotein constructs, including flanking I27, were synthesised and cloned into the pFN18a vector. The polyproteins were expressed in *Escherichia coli* BL21-CodonPlus (DE3)-RILP competent cells, using the T7 promoter present in the plasmid. Protein expression was induced with IPTG when the culture reached an OD600 nm of 0.6. Cells were lysed with lysozyme and sonication before protein purification with Ni-NTA beads in a batch process. The eluted proteins were analysed for purity with SDS-PAGE. Final concentration of protein used for experiments was 1–10 μg/ml. Glass coverslips were functionalised with the chloroalkane ligand to HaloTag as previously described [32]. The glass coverslips were first cleaned using Helmanex III (1% in water), acetone, and ethanol washes. The surfaces were then prepped with O2 plasma cleaning for 15 min. Surfaces were then silanised using (3-aminopropyl)trimethoxysilane diluted to 1% in ethanol. Surfaces were then washed with ethanol and then dried with N2. These amine-functionalised surfaces were then incubated with 10 mM succinimidyl-[(Nmaleimidopropionamido)tetracosaethyleneglycol] ester (SMPEG24, Thermo) diluted in 100 mM borax buffer (pH 8.5) for 1 h. The final step involved incubating the surfaces overnight with 10 mM HaloTag Thiol O4 ligand in the same buffer. The surfaces were quenched 50 mM 2-mercaptoethanol in water. We used a commercial AFS-1 from Luigs & Neumann, GmbH, based on a device developed at the Fernandez Lab, Columbia University [33]. The cantilevers used were gold-coated OBL-10 levers from Bruker. The spring constants varied between 4–10 pN/nm as measured by equipartition theorem with the appropriate adjustments for cantilever geometry. Around 20 μl of protein solution was incubated on functionalised coverslips for 30 min prior to the experiments to allow for HaloTag binding. The cantilever was pressed into the surface with a force of approximately 300 pN to bind the cantilever to the polyprotein. Force extension experiments were conducted at 400 nm/s retraction rate. Data analysis was carried out using Igor Pro (Wavemetrics), for which the wormlike chain model was applied.

### SMD simulations

The following structures from RCSB Protein Data Bank were used as the protein models for the individual talin rod subdomains: R8 (id 5FZT residues 1451–1588), R8-DLC1 (id 5FZT talin residues 1451–1588 and DLC1 residues 467–489), and R7R8 (id 2X0C). The talin structures were modified in PyMOL to produce the desired constructs.

MD and SMD simulations were performed using Gromacs ver 2016.1 [34, 35] at the Sisu supercomputer, CSC, Finland. The CHARMM27 force field [36] and explicit TIP3P water model [37] in 0.15 M KCl solution were used, and the total charge of the system was adjusted by K+ and Cl− ions. The energy minimisation of the system was performed in 100,000 steps, using the steepest descent algorithm.

The equilibration was performed with NPT ensemble and was maintained at 310 K using the V-rescale algorithm [38] and 1 atm under Berendsen barostat, as implemented in Gromacs 2016.1. First run of equilibration was performed for R8 and R8-DLC1 with long linkers. These complexes were followed over 30 ns simulation without applied restrictions. Both domains were very stable, with low root-mean-square deviation (RMSD) changes. Major structural changes were observed for the long interdomain linker. In the second equilibration run, the fixed and pulled Cα atoms were restrained with harmonic potential during the 10 ns simulation. The temperature coupling was applied separately for the protein and the solution parts. Each system was equilibrated up to 10 ns, with subsequent monitoring of the RMSDs of Cα atoms. Structures after 10 ns were used as the starting pulling conformation. Pulling vector was set between Cα of the first and the last residue of the appropriate domain parallel to the z-axis of the pulling box. The movement of Cα of N-terminal residue was restrained with harmonic potential, while Cα of C-terminal residue was subjected to the constant velocity pulling. After placing the equilibrated protein in the pulling box and orienting it appropriately in the pulling direction, the water solution was equilibrated as external bath during 1 ns bath equilibration [39]. Furthermore, equilibration was performed at isotropic conditions while pulling at semi-isotropic conditions when pressure control was turned off in the pulling direction (z-axis). The constant velocity pulling SMD simulations were performed at 2 nm/ns with the spring constant set to 1,000 kJ/mol nm^2^. All trajectories were produced in 3 repetitions.

### Cells and culture conditions

The WT MEF cell line was a kind gift from Dr Wolfgang Ziegler and has been previously described by Xu and Baribault in 1998 [40]. The *Tln1−/−Tln2−/−* MEF cell line has been previously described by Theodosiou and colleagues [17]. Both cell lines were maintained in high-glucose DMEM supplemented with 10% FBS and 1% GlutaMax (Thermo Fisher Scientific, United States of America). A humidified 37°C incubator with 5% CO_2_ was used for culturing both cell lines. Cells were negative when tested for mycoplasma contamination.

### Plasmid constructs for use in MEFs

In all cell experiments other than the FRAP, a GFP-Talin1 plasmid was used. The GFP-Talin1 plasmid was a gift from Anna Huttenlocher (Addgene plasmid # 26724) [41]. The GFP-DLC1 plasmid was a gift from Irene Ng [42]. The GFP-vinculin plasmid was obtained from Susan Craig [43]. The GFP-paxillin was a gift from Chinten James Lim (Addgene plasmid # 80023). The talin 1 plasmid (Addgene # 26724) was used as template to generate the R8, C1, and C2 constructs by PCR-based mutagenesis starting construct via 5′ FseI and 3′ Scal. R8: For the substitution of amino acid glutamine with cysteine at position 1459, CAA was replaced with TGC; and for the substitution of serine for cysteine at position 1583, AGC was replaced with TGC. C1: For the substitution of amino acid glutamine with cysteine at position 1459, CAA was replaced with TGC. C2: For the substitution of serine for cysteine at position 1583, AGC was replaced with TGC.

### FRAP

The GFP-DLC1 FRAP experiments were conducted on glass-bottom petri dishes (Mattek) coated with human plasma FN (10 μg ml^−1^; Sigma) and incubated at 37°C. For the DTT condition, DTT (Sigma) was added at a concentration of 100 μM 30 min prior to the start of the experiment. Confocal photobleaching and TIRF imaging were carried out using an inverted microscope (Eclipse Ti; Nikon). Five TIRF images were taken at 5 s intervals prior to bleaching for reference. Specified regions of the cells were then bleached using the confocal laser at 100% power. TIRF images were taken at 5 s intervals for 100 s to capture fluorescent recovery. Images were analysed with FIJI, with the fluorescent signal normalised between the prebleach intensity and background. Statistical analysis was then carried out using Prism (GraphPad). Data were pooled from repeats. The significance between curves was measured using extra sum-of-squares F test on the best fit lines.

### MLC-2 immunostaining

Immunofluorescence staining was done on coverslips coated with 10 μg/ml fibronectin (Sigma, F0895). Following pertinent treatment, cells were fixed with 4% PFA (Sigma, P6148) in PBS for 10 min and then blocked and permeabilised with 2% BSA-0.1%Triton (Sigma, T8787) in PBS for 30 min. After blocking, cells were incubated with primary antibodies (MLC-2 Millipore MABT180 1/200 and pMLC-2 Thr18/Ser19 Cell Signalling 3674 1/200) prepared in blocking solution for 1 h at room temperature in a humidified chamber. Then cells were washed in PBS and incubated with Alexa Fluor 488 conjugated secondary antibodies and Phalloidin (Invitrogen, A22283, 1/1,000 dilution) prepared in PBS for 30 min at room temperature. Finally, coverslips were washed in PBS and mounted in mounting reagent with DAPI (Invitrogen, P36931). GSH was purchased from Sigma-Aldrich (catalogue 78259) and used at a concentration of 10 mM. DLC1 antibody (clone C-12, catalogue sc-271915, dilution 1/100). siRNA for DLC1 was from Santa Cruz Biotechnology, catalogue sc-72134. Primers used to monitor DLC1 gene expression knockdown: DLC1 (m)-PR, catalogue sc-72134-PR.

### Micropillar video microscopy and traction force measurements

The micropillar arrays are based on our protocol as described previously [22]. Pillar arrays were coated with human plasma FN (10 μg ml^−1^; Sigma) and incubated at 37°C for 1 h before measurements. Cells that had been trypsinised before measurements were suspended in culture media and plated onto the pillar substrates. Time-lapse imaging of the pillars was conducted with an inverted microscope (Eclipse Ti; Nikon) operating in bright-field mode, with the samples held at an ambient temperature of 37°C. Image sequences were recorded with a sCMOS camera (Neo sCMOS Andor) at 0.5 Hz using a ×40 (0.6 NA, air; Nikon) objective over 100 min. The position of each pillar in the time-lapse videos was tracked using a custom MATLAB program to track the centre of a point spread function of the intensity of the pillars across all frames. By selecting a location free of cells, tracking of a small set of pillars allowed a measurement of the stage drift to be obtained and corrected for in the data set. The time-dependent displacement of a given pillar was obtained by subtracting the initial position of the pillar (zero force) from the position in a given frame. Traction forces were obtained by multiplying the pillar displacements by the pillar stiffness; the maxima for each pillar were found to obtain the peak forces across the cell.

### Gel contraction

To analyse the ECM remodelling ability of MEFs, collagen-I (BD Biosciences, 354249, stock concentration 9.37 mg/ml) and Matrigel (BD Biosciences, 354234, stock concentration 9 mg/ml) mixture gels were prepared with 10% 10× DMEM (Sigma, D2429) and 10% FBS (Gibco, 10500), yielding to a final concentration of 4.4 mg/ml collagen-I and 2.2 mg/ml Matrigel. The gel mixture was neutralised with 1 M NaOH (Sigma, S8045); then, 5 × 10^5^ cells were embedded in gels in culture media. Eighty μl gel volume was added per well of a 96-well plate, which was pretreated with 2% BSA (Sigma, A8022) for 1 h, washed with PBS, and air dried for 10 min. Gels were set 1 h at 37°C and then incubated with culture media for 2 d at 37°C.

### Migration assays

Polystyrene 6-well plates were coated with 10 μg/ml fibronectin in PBS (pH 7.4) at 37°C for 1 h and washed 2 times with PBS. Transfected *Tln1−/−Tln2−/−* MEF cells were allowed to recover for 24 h, trypsinised, and plated onto fibronectin-coated well plates at a low confluency. Cells were allowed to attach for 90 min, followed by replacement of media (2 ml of fresh media) to remove nontransfected cells. For treating the cells with diamide, diamide powder (Sigma-Aldrich D3648) was dissolved in PBS to a stock concentration of 50 mM (8.6 mg/ml) and further diluted with complete cell culture media to 150 μM concentration. One ml of the diamide-containing media was added dropwise to 2 ml of cell culture media in each well 15 min before live cell imaging was started. EVOS FL Auto (Life Technologies, USA) equipped with a 20× objective and 37°C and 5% CO_2_ incubator was used for live cell imaging for 12 h at 120 s intervals. The resulting image stacks were analysed with ImageJ version 1.50e with MTrackJ plugin [44, 45]. For each plasmid construct or diamide treatment, 130–200 individual cells were traced from 3 fully independent experiments.

### 2D SDS-PAGE analysis

Lysates of diamide-treated WT MEF cells were analysed by 2D SDS-PAGE to confirm the effect of diamide treatment on the level of cellular disulphide bonds. Cells at 80% confluency on 10 cm dishes were washed twice with warm PBS and treated for 120 min with 50 μM diamide dissolved in cell culture media. In negative controls, regular media were used instead. After the diamide treatment, cells were washed with ice-cold PBS and treated with 40 mM iodoacetamide in PBS for 5 min. Cells were lysed with 500 μl of RIPA buffer (50 mM Tris-HCl pH 7.4, 1% NP-40, 0.5% Na-deoxycholate, 0.1% SDS, 150 mM NaCl, 50 mM NaF) supplemented with 40 mM iodoacetamide and Roche complete protease inhibitor cocktail. Cell lysates were incubated on ice for 30 min and centrifuged at 14,000 g for 20 min at 4°C to pellet cell debris. The concentrations of cleared lysates were determined with BCA assay and matched by diluting the lysates with RIPA buffer. For 2D SDS-PAGE, 120 μg samples of cell lysates were mixed with 2× Laemmli sample buffer without reducing agents, denatured at 95°C for 5 min, and loaded onto a medium-sized 1 mm thick 10%/4% polyacrylamide gel. The polyacrylamide gels were run at 25 mA current for 5 h, followed by vertical slicing of the gel with a clean scalpel to separate each lane. The gel slices were incubated in SDS-PAGE running buffer supplemented with 100 mM DTT for 20 min at room temperature to reduce disulphide bonds in the lysates. After the treatment, the gel slices were briefly washed with running buffer and treated with 100 mM iodoacetamide in SDS-PAGE running buffer for 10 min. The pieces of gel were placed horizontally on top of 1.5 mm thick 10% polyacrylamide gels and ran at 10 mA current for 14 h. Proteins on the gel were visualised by using silver staining.

### Statistical analysis

All statistical analyses were conducted with the Prism graphical software (GraphPad, Software). Data were generated from multiple repeats of different biological experiments in order to obtain the mean values and standard errors (sem) displayed throughout. *P*-values have been obtained through *t* tests. Significance for the *t* tests was set at *P* < 0.05, for which graphs show significance through symbols (*, *P* < 0.05; **, *P* < 0.01; ***, *P* < 0.001; ****, *P* < 0.0001).

## Supporting information

S1 Data Raw numerical values for all quantitative data. This file has the raw numbers for the following figure panels: Figs 2C and 2E, 4B, 4C, 4D, 4E, 4F, 4G, 4H and 4J, 5B, 5C, 5D and 5F.
(XLSX)

S1 Fig Unfolding geometry and unfolding force demand. The hypothetical comparison of 4-helix and 5-helix bundles’ mechanical stability under end-to-end pulling.
(PDF)

S2 Fig Comparison of bleached to nonbleached regions of MEF cells in FRAP. (A) Representative images of a cell region exposed to confocal laser during FRAP, demonstrating photobleaching and subsequent recovery. (B) Images of a region of the same cell not exposed to high-power confocal laser, displaying no apparent photobleaching. Scale bar 5 μm. FRAP, fluorescent recovery after photobleaching; MEF, mouse embryonic fibroblast.
(PDF)

S3 Fig FRAP data for DLC1 GFP. Representative images of a cell region exposed to confocal laser during FRAP, demonstrating photobleaching and subsequent recovery. WT: WT talin, R8: clamped R8 domain in talin, C1: talin control with 1 cysteine in position 1459 of the amino acid sequence, C1: talin control with 1 cysteine in position 1583 of the amino acid sequence. Scale bar is 5 μm. These images correspond to Fig 4 panels B-E. DLC1, deleted in liver cancer 1; DTT, dithiothreitol; FRAP, fluorescent recovery after photobleaching; GFP, green fluorescent protein; WT, wild-type.
(PDF)

S4 Fig FRAP data for vinculin GFP (two upper rows) and paxillin GFP (two lower rows). Representative images of a cell region exposed to confocal laser during FRAP, demonstrating photobleaching and subsequent recovery. WT: WT talin, R8: clamped R8 domain in talin. Scale bar is 5 μm. These images correspond to Fig 4 panels F-H. FRAP, fluorescent recovery after photobleaching; GFP, green fluorescent protein; WT, wild-type.
(PDF)

S5 Fig Knockdown efficiency in the DLC1 siRNA cells. PCR levels of DLC1 expression in Tln1−/−Tln2−/− MEF cells transfected with WT and R8 talin constructs. Values are normalised to GAPDH and relative to control. Histogram bars represent mean ± sem, ***P < 0.001, (*t* test). Three experimental replicates. DLC1, deleted in liver cancer 1; GAPDH, glyceraldehyde 3-phosphate dehydrogenase; MEF, mouse embryonic fibroblast; siRNA, short interfering RNA; WT, wild-type.
(PDF)

S6 Fig 2D SDS-PAGE analysis of proteins in talin-null MEF cells after treatment with 50 μM diamide revealed a clear increase in the level of disulphide bonds. To confirm the increase in the level of cellular disulphide bonds after a treatment with 50 μM diamide, lysates of diamide-treated MEF cells were analysed with 2D SDS-PAGE. Proteins in total-cell lysates were first separated without reducing agents (horizontal axis), followed by excising each gel lane to separate pieces and treating the gel lanes with DTT to reduce the disulphide bonds. The proteins on the reduced lanes were separated in a second SDS-PAGE run in the other direction to reveal the differential migration of disulphide-containing proteins in oxidising and reducing conditions. Proteins that do not contain disulphide bonds migrate at similar rates in both gel runs and end up on a diagonal band on the gel. (A) Intra- or intermolecular disulphide bonds affect the rate of protein migration on gel, typically slowing down the rate of protein migration on the gel. Thus, proteins with intra- or intermolecular disulphide bonds migrate at a faster rate after breaking the disulphide bonds by reducing conditions and appear below the diagonal line at the 2D SDS-PAGE analysis. (B) The images presented are representative of two fully independent replicates. DTT, dithiothreitol; MEF, mouse embryonic fibroblast.
(PDF)

S1 Text Analysis of molecular dynamics simulation data in Fig 3. In the main text, the analyses of the SMD are summarised and presented in full in the section S1 Text. SMD, steered molecular dynamics.
(DOCX)

S1 Video Migration of talin-null MEFs transfected with WT and R8 (in no diamide condition and when 50 μm of diamide was added). Fifteen frames per second. Scale bar 100 μm. MEF, mouse embryonic fibroblasts; WT, wild type.
(AVI)

## Abbreviations

a.u.: arbitrary unit
DLC1: deleted in liver cancer 1
DTT: dithiothreitol
ECM: extracellular matrix
FRAP: fluorescent recovery after photobleaching
FRET: Förster resonance energy transfer
GFP: green fluorescent protein
GSH: glutathione
MEF: mouse embryonic fibroblast
MFI: mean fluorescence intensity
MLC-2: myosin light chain 2
PDMS: polydimethylsiloxane
RhoA: Ras homolog family member A
RhoGAP: Ras homolog GTPase-activating protein
RMSD: root-mean-square deviation
smAFM: single-molecule atomic force microscopy
SMD: steered molecular dynamics
SMPEG24: succinimidyl-[(Nmaleimidopropionamido)tetracosaethyleneglycol] ester
WT: wild-type
