# Supplementary figures and images for: Mechanotransduction in talin through the interaction of the R8 domain with DLC1
Source: PLoS Biol. 2018 Jul 20;16(7):e2005599. doi: 10.1371/journal.pbio.2005599 (PMC6054372; doi:10.1371/journal.pbio.2005599)

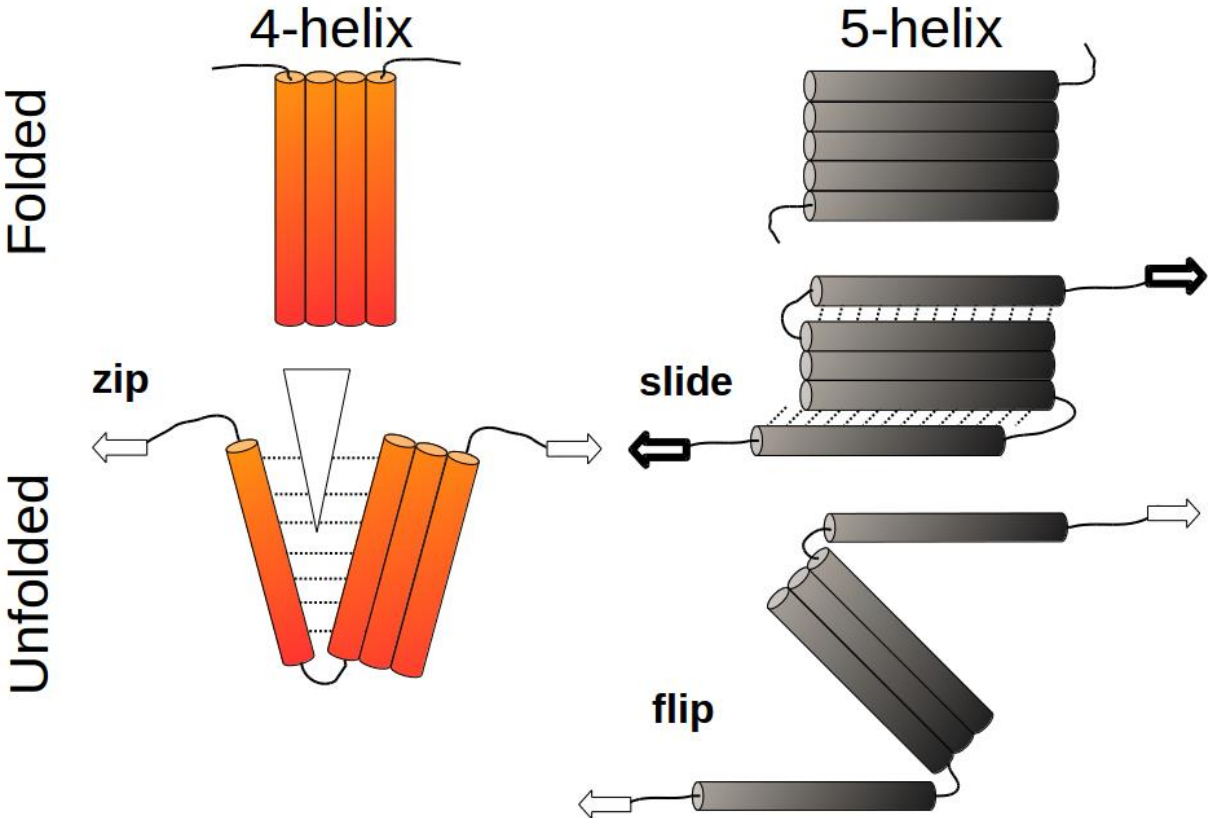

Supplement: S1 Fig — The hypothetical comparison of 4-helix and 5-helix bundles’ mechanical stability under end-to-end pulling. (PDF) [file pbio.2005599.s002.pdf]

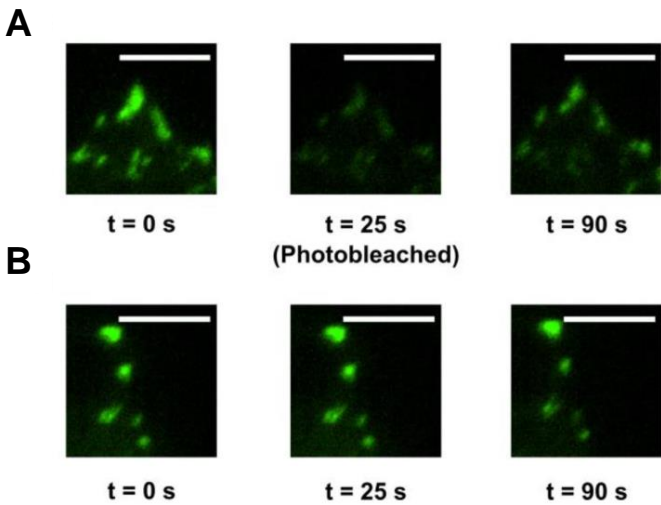

Supplement: S2 Fig — (A) Representative images of a cell region exposed to confocal laser during FRAP, demonstrating photobleaching and subsequent recovery. (B) Images of a region of the same cell not exposed to high-power confocal laser, displaying no apparent photobleaching. Scale bar 5 μm. FRAP, fluorescent recovery after photobleaching; MEF, mouse embryonic fibroblast. (PDF) [file pbio.2005599.s003.pdf]

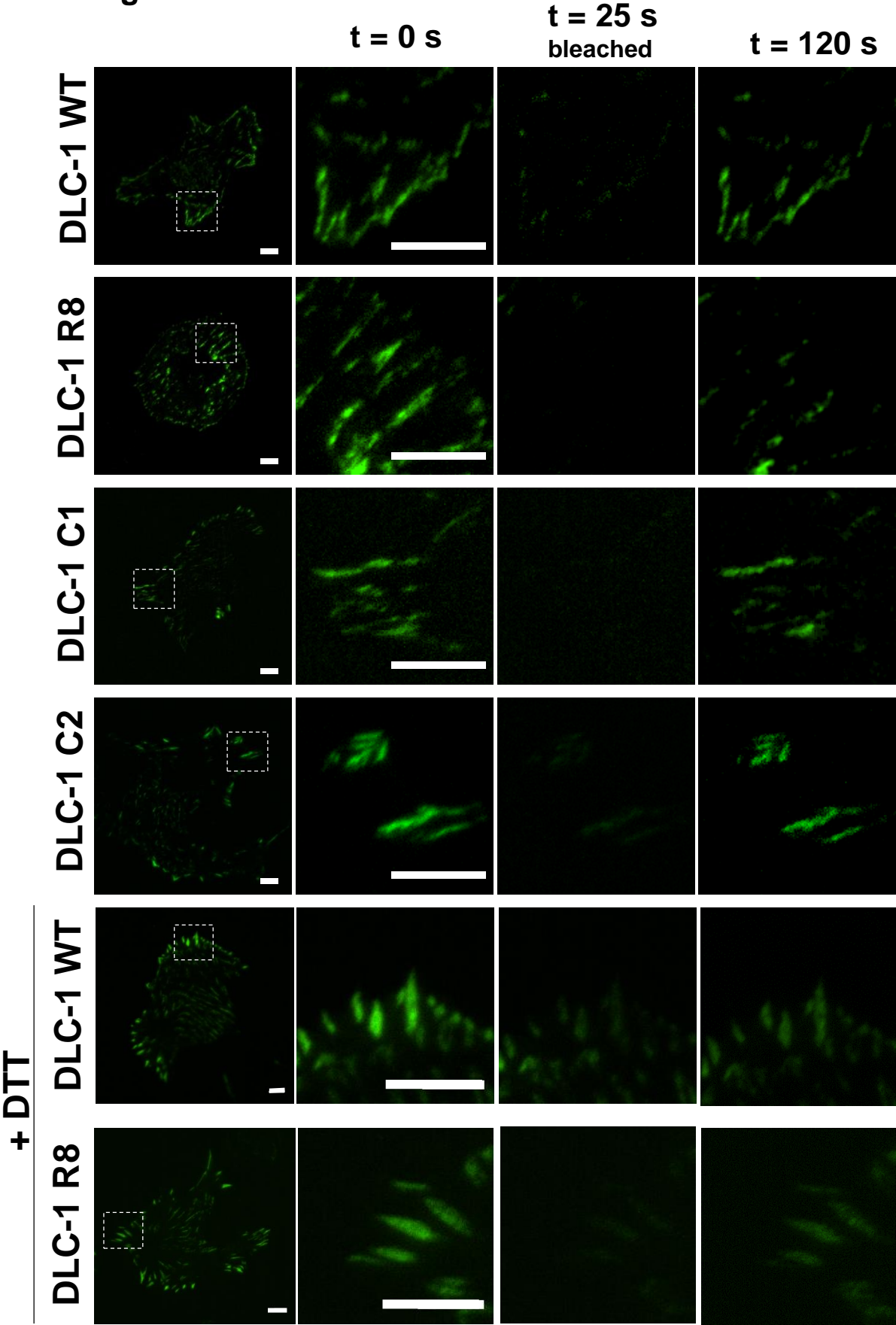

Supplement: S3 Fig — Representative images of a cell region exposed to confocal laser during FRAP, demonstrating photobleaching and subsequent recovery. WT: WT talin, R8: clamped R8 domain in talin, C1: talin control with 1 cysteine in position 1459 of the amino acid sequence, C1: talin control with 1 cysteine in position 1583 of the amino acid sequence. Scale bar is 5 μm. These images correspond to Fig 4 panels B-E. DLC1, deleted in liver cancer 1; DTT, dithiothreitol; FRAP, fluorescent recovery after photobleaching; GFP, green fluorescent protein; WT, wild-type. (PDF) [file pbio.2005599.s004.pdf]

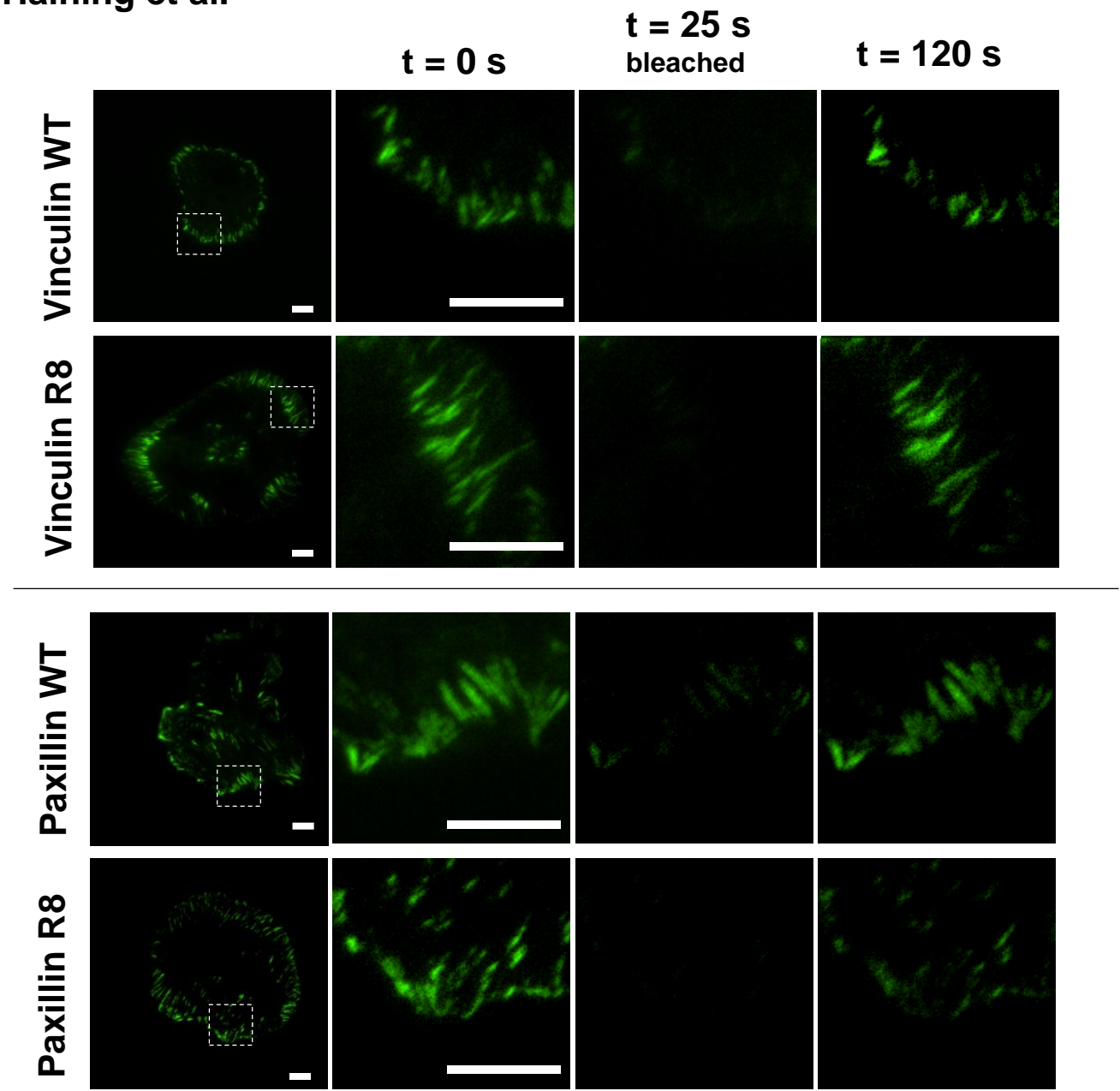

Supplement: S4 Fig — Representative images of a cell region exposed to confocal laser during FRAP, demonstrating photobleaching and subsequent recovery. WT: WT talin, R8: clamped R8 domain in talin. Scale bar is 5 μm. These images correspond to Fig 4 panels F-H. FRAP, fluorescent recovery after photobleaching; GFP, green fluorescent protein; WT, wild-type. (PDF) [file pbio.2005599.s005.pdf]

Haining et al.

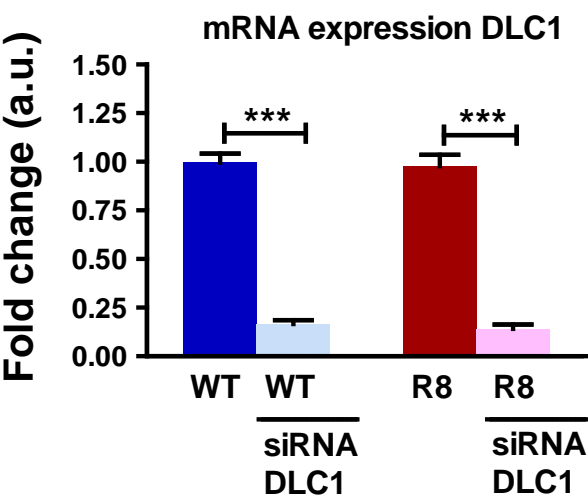

Supplement: S5 Fig — PCR levels of DLC1 expression in Tln1−/−Tln2−/− MEF cells transfected with WT and R8 talin constructs. Values are normalised to GAPDH and relative to control. Histogram bars represent mean ± sem, ***P < 0.001, (t test). Three experimental replicates. DLC1, deleted in liver cancer 1; GAPDH, glyceraldehyde 3-phosphate dehydrogenase; MEF, mouse embryonic fibroblast; siRNA, short interfering RNA; WT, wild-type. (PDF) [file pbio.2005599.s006.pdf]

A) 0  $\mu$ M Diamide      ← Oxidizing (1)

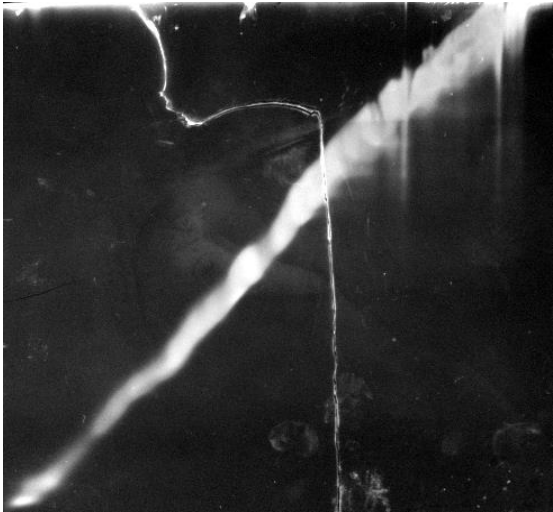

B) 50  $\mu$ M Diamide      ← Oxidizing (1)

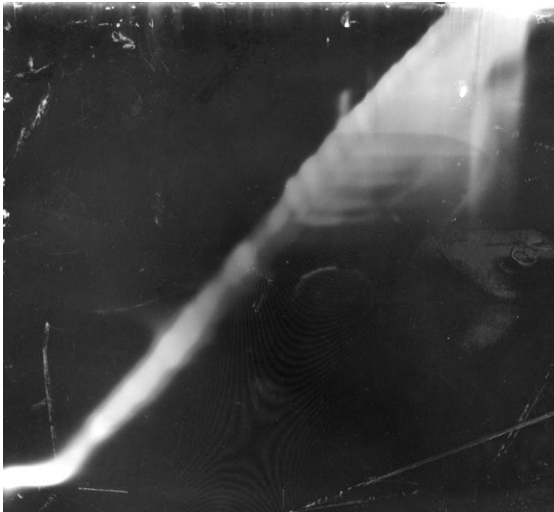

Supplement: S6 Fig — To confirm the increase in the level of cellular disulphide bonds after a treatment with 50 μM diamide, lysates of diamide-treated MEF cells were analysed with 2D SDS-PAGE. Proteins in total-cell lysates were first separated without reducing agents (horizontal axis), followed by excising each gel lane to separate pieces and treating the gel lanes with DTT to reduce the disulphide bonds. The proteins on the reduced lanes were separated in a second SDS-PAGE run in the other direction to reveal the differential migration of disulphide-containing proteins in oxidising and reducing conditions. Proteins that do not contain disulphide bonds migrate at similar rates in both gel runs and end up on a diagonal band on the gel. (A) Intra- or intermolecular disulphide bonds affect the rate of protein migration on gel, typically slowing down the rate of protein migration on the gel. Thus, proteins with intra- or intermolecular disulphide bonds migrate at a faster rate after breaking the disulphide bonds by reducing conditions and appear below the diagonal line at the 2D SDS-PAGE analysis. (B) The images presented are representative of two fully independent replicates. DTT, dithiothreitol; MEF, mouse embryonic fibroblast. (PDF) [file pbio.2005599.s007.pdf]
