# Supplementary material for: Mechanotransduction in talin through the interaction of the R8 domain with DLC1
Source: PLoS Biol. 2018 Jul 20;16(7):e2005599. doi: 10.1371/journal.pbio.2005599 (PMC6054372; doi:10.1371/journal.pbio.2005599)
Supplement: S1 Text — In the main text, the analyses of the SMD are summarised and presented in full in the section S1 Text. SMD, steered molecular dynamics. (DOCX) [file pbio.2005599.s008.docx]

**S1 Data: Full description of the SMD data presented in figures 3 and S1.**

In molecular dynamics simulations, the DLC1 binding to the R8 domain did not show pronounced changes in the domain conformation over 10 and 30 ns molecular dynamics simulation as shown by the structural alignment between the structure R8 (id 5FZT) and R8 in R8-DLC1 complex (id 5FZT) after 10ns equilibration. Indeed, the R8 alone as well as in the DLC1 complex are stable conformations. All-atom RMSD between the two structures was 0.83Å. A 30 ns molecular dynamics simulation resulted in a value of 0.89Å for all-atom RMSD between the R8 subdomains **(Fig. 3A).** Talin helices, namely R8 H2H3 facilitating the DLC1 binding showed RMSD of 0.84Å after 30 ns equilibration**.**

Steered molecular dynamics simulation was performed to test the mechanical stability and properties of the R7-R8 double domain structure. In the 2nm/ns constant velocity pulling simulation, the unfolding of the 5-helix R7 domain occurred between 1 and 2ns of the simulations at approximately 400pN force magnitude **(Fig. 3B).** The R7 domain break was consistent over the three repetitions considering the time as well as the force magnitude. The 4-helix R8 domain unfolding was observed at distinctly lower force compared to the R7 domain at approximately 200pN. Greater variation in the time of the R8 domain break was detected over the three repetitions. This may be due to the complexity of the simulation system and number of possible domain orientations over the pulling simulation.

This force magnitude required to unfold R8 is comparable to the force needed to unfold talin R3 domain in SMD simulations[1]. In experimental setting, R3 and R8 domains are considered mechanically weak talin domains[2, 3].

Furthermore, we have recognized the stable 3H intermediate state as described in our previous work[1]. We have characterized the 3H state being generally slightly less mechanically stable compared to the 5-helix bundle. In the force traces shown in **Fig. 3B**, the 3H state appears to break at approximately at 500pN of force magnitude. This increased force demand is possibly due to other unfolding resisting events and bonds taking place in this complex double domain system. From the R7-R8 force trace plot **(Fig. 3B)** a significant force increase is observed between 5ns and 17ns in all the repetitions. This corresponds to a conformation, where the unfolded R7 H1 helix creates contact with the intact R8 domain, i.e. a helix swap state. The existence of this state and its biological roles remain to be studied experimentally.

We also investigated the effect of the DLC1 binding on the mechanical stability of R8 domain **(Fig. 3C).** We examined the R8 domain including the linkers connecting it to the R7 domain and R8-DLC1 complex including the linker with identical N- and C-terminal residues in constant velocity SMD simulation at 2nm/ns speed. **Fig. 3C** presents the force traces for both investigated R8 domains (three parallel repetitions for each R8 and R8-DLC1). Based on the SMD results we conclude that DLC1 binding does not affect the mechanical stability of R8 domain. The domain break is observed at similar time over the trajectories and at a similar force magnitude. In the unfolding of the R8-DLC1 complex, we identified one additional peak in the force trace corresponding to the unfolding of the R8 H2H3-DLC1 complex, here marked as “C”. This force magnitude is comparable to the force needed to unfold the 4-helix R8 domain. Based on our previous findings, this force peak resulting from the unfolding of the R8 H2H3-DLC1 complex would remain undetected in the experimental setup.

**References for S1 Text**

1. Mykuliak VV, Haining AWM, von Essen M, Del Rio Hernandez A, Hytonen VP. Mechanical unfolding reveals stable 3-helix intermediates in talin and alpha-catenin. Plos Comput Biol. 2018;14(4):e1006126. doi: 10.1371/journal.pcbi.1006126. PubMed PMID: 29698481.

2. Haining AWM, von Essen M, Attwood SJ, Hytönen VP, del Río Hernández A. All Subdomains of the Talin Rod Are Mechanically Vulnerable and May Contribute To Cellular Mechanosensing. ACS Nano. 2016. doi: 10.1021/acsnano.6b01658.

3. Yao MX, Goult BT, Klapholz B, Hu X, Toseland CP, Guo YJ, et al. The mechanical response of talin. Nat Commun. 2016;7. doi: ARTN 11966

10.1038/ncomms11966. PubMed PMID: WOS:000380302500001.
